# Threading dislocation reduction in heteroepitaxial GaSb based superlattices grown on silicon

**DOI:** 10.1038/s41598-026-55373-4

**Published:** 2026-06-03

**Authors:** Evangelia Delli, Mathew Bentley, Niall Mulholland, Peter D. Hodgson, Maria de la Mata, Sergio I. Molina, Richard Beanland, Magnus C. Wagener, Johannes R. Botha, Andrew R. J. Marshall, Peter J. Carrington

**Affiliations:** 1 School of Engineering, Lancaster University, Bailrigg, Lancaster, LA1 4YW UK; 2 Department of Physics, Lancaster University, Bailrigg, Lancaster, LA1 4YB UK; 3 Department of Material Science and Metallurgic Engineering, University of Cadiz, Puerto Real, Cadiz, 11510 Spain; 4 Physics Department, University of Warwick, Coventry, CA4 TAL UK; 5 Department of Physics, Nelson Mandela University, Gqeberha, 6031 South Africa

**Keywords:** Optical materials and structures, Lasers, LEDs and light sources

## Abstract

Direct epitaxial growth of gallium antimonide (GaSb) on silicon (Si) holds the potential for creating new applications in mid-infrared (MIR) silicon photonics. However, this is challenging due to the significant physical dissimilarities between the two materials creating various crystal imperfections and defects. The main challenge is the generation of threading dislocations arising from the high lattice mismatch. To successfully implement GaSb-based devices on silicon a major engineering breakthrough is required to significantly reduce the dislocation density. This work presents a study of high quality GaSb epilayers on Si with defect density of 6 × 10^6^ cm^-2^ grown using molecular beam epitaxy (MBE). This was achieved using a novel growth procedure consisting of an efficient AlSb interfacial misfit array, a two-step GaSb growth temperature procedure and strained dislocation filter superlattices (DFSLs). The superlattice (SL) layers were carefully engineered and positioned to prevent the generation of new defects while blocking the vertical propagation of pre-existing ones. The successful reduction of the threading dislocations and excellent crystalline quality was identified through a significant increase of the photoluminescence intensity.

**Supplementary Information:**

The online version contains supplementary material available at 10.1038/s41598-026-55373-4.

## Introduction

Silicon (Si) based photonic integrated circuits have emerged as a promising platform for realizing single-chip photonic systems. The main advantages of this technology are the low material losses and the mature complementary metal-oxide semiconductor (CMOS) compatible fabrication technology which enables low-cost and wafer-scale manufacturing. A wide range of passive optical components have been implemented on Si including high speed modulators^1^, low loss waveguides^2^ and grating couplers with potential applications in gas sensing^3^, biomedical diagnostics^4^ and data communications^5^. However, wafer level integration of efficient light sources is still hindered by the indirect band gap of Si and Si-based materials, which limits the realization of complex on-chip passive and active functionalities. One of the most promising solutions is the direct epitaxial growth of III-V semiconductor materials on Si. The key advantages of III-V materials are their high carrier mobilities and absorption coefficients, and their direct band gaps. Among the III-Vs, gallium antimonide (GaSb) and its alloys are the present materials of choice for applications in the mid-infrared (MIR) spectral region such as environmental, industrial and medical sensing. Therefore, the growth of high quality GaSb on Si would extend the operating wavelength of Si photonics towards the MIR to enable on-chip, compact optoelectronic systems for real-time gas monitoring, lab-on-a-chip applications and monolithic integration of focal plane arrays (FPAs) with Si readout integrated circuits (ROICs). Nevertheless, the large material dissimilarities between GaSb and Si lead to the formation of defects which degrade the optical device performance and reliability. The main challenges are the high lattice mismatch (12.2%) leading to the generation of a very high density of threading dislocation (TDs)^6,7^ and the polar/non-polar character of the III-V/Si interface which induces antiphase domain (APDs)^8^ defects. Furthermore, the potential formation of thermal cracks^9^ due to the large differences in the thermal expansion coefficient between the Si wafer and the III-V semiconductor epilayer may also restrain the usefulness of this technology.

The use of offcut Si wafers (4 to 6 degrees), characterized by a high density of bi-atomic steps on the surface^10^, alongside the initial formation of a Si-V interface across the entire wafer is a widespread strategy used for limiting the formation of APDs in III-Vs deposited on Si. High temperature annealing of the substrate prior to the heteroepitaxial growth has also been seen to promote the formation of well-ordered surface steps along the offcut direction^11,12^. This can effectively bury the APDs formed at the interface within the first few nanometers of the III-V growth, even when low offcut Si wafers (< 0.2 degrees) are used. The use of a thin, annealed Si buffer layer prior to the III-V deposition also promotes the periodic ordering of the atomic steps with different dimerization on the Si surface^12^. Even so, the formations of interfacial misfit dislocation arrays (IMF) and TDs is inevitable as the generation and movement of dislocations is a consequence of relieving the high interfacial misfit strain and total energy of the system. While the misfit dislocations are accommodated across the III-V epitaxial film and the substrate, the TDs can propagate through the entire epitaxial structure, reaching defect densities of the order of 10^9^– 10^10^ cm^− 2^. The inability to control their propagation is detrimental to the performance of optical devices, as the defects are associated with the presence of non-radiative recombination centers^13^. A range of techniques have been used to reduce the TD density, including annealing assisted epitaxial growth^14^ and selective area heteroepitaxy^15,16^. One of the most popular methods used for III-V buffer layers grown on Si is the growth of several strained layer dislocation filter superlattices (DFSLs)^17^ usually separated by spacer layers. These structures consist of several layers with alternating compressive/tensile misfit strain at the interfaces. The strain fields exert a lateral force on the threading dislocations, causing their glide along the interface. As a result, the dislocations are eliminated through interactive encounters with others or escape at the edge of the sample. However, the dislocations that do not glide sufficiently to interact will propagate within the structure or be replicated in the next interface leading to longer line threading lengths. By utilizing several strained superlattices (SLs) as a barrier to the vertical propagation of dislocations, an efficient heteroepitaxial III-V/Si substrate structure can be achieved^17^, potentially replacing the native wafers traditionally used in III-V semiconductors epitaxy.

Several authors^18,19^ have reported substantial device improvement and long-term reliability^20,21^ for GaAs on silicon devices by lowering the threading dislocation density down to levels of 10^6^ cm^− 2^. Recently, it was suggested that to achieve a similar threshold current for mid-infrared lasers grown on GaSb/Si buffer layers to those obtained for homoepitaxial devices, a defect level of a few 10^6^ cm^− 2^ needs to be reached^22^. However, so far, the lowest reported TD density for GaSb on Si falls within the range of 1 to 5 × 10^7^ cm^[− 2 23,24^. This work reports on the heteroepitaxial growth of GaSb epilayers on Si with a record surface dislocation density of 6 × 10^6^ cm^− 2^ using strained DFSLs and presents the structural and optical properties achieved.

### Epitaxial growth and DFSL structure design

**Fig. 1:**
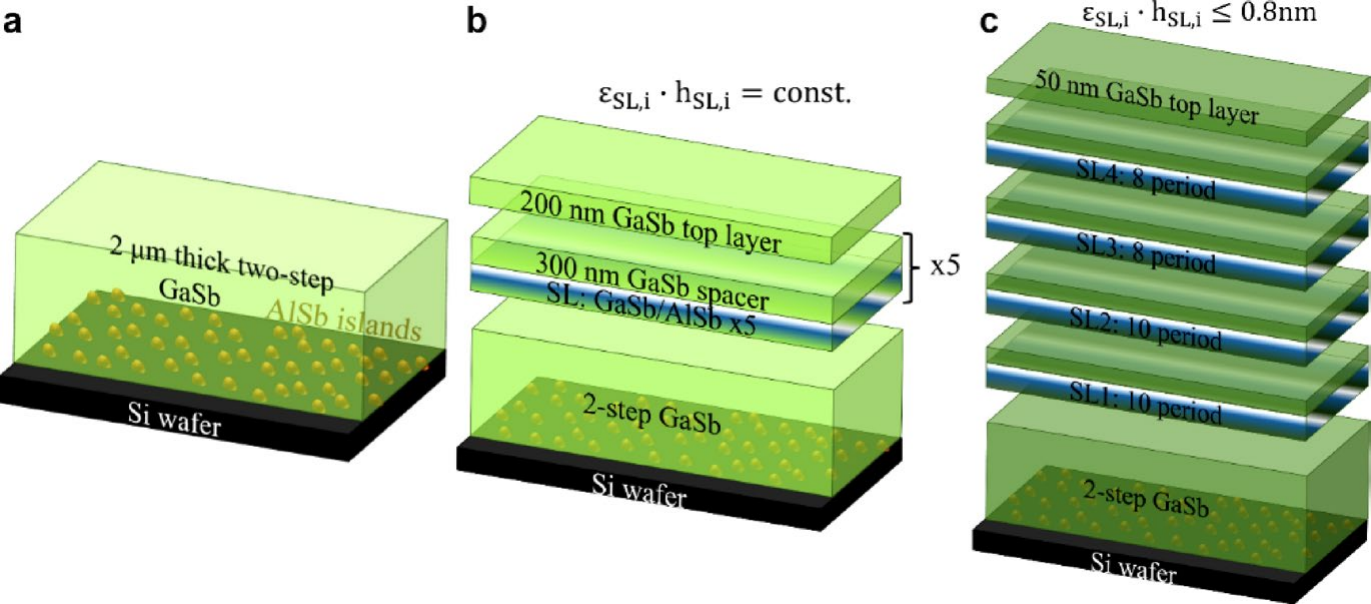
Schematic diagram of the three samples presented in this work: (**a**) The 2 μm thick, two-step GaSb buffer sample. (**b**) SL sample A with a total thickness of 4.2 μm, consisting of five identical AlSb/GaSb DSFLs, each having a strain-thickness product 0.325 nm. (**c**) SL sample B with a total thickness of 3.7 μm, consisting of four AlSb/GaSb DSFLs, each having different strain-thickness products, as outlined in Table 1. A 200 nm GaSb spacer layer was used between each SL.

Three samples were grown directly on Si substrates with 4° miscut towards the [0–11] direction using molecular beam epitaxy (MBE). Removal of the surface silicon oxides was performed in situ using a stepped heating procedure lasting more than one hour, reaching a maximum annealing temperature of roughly 1000 °C. Following the oxide desorption, the wafers were cooled down to the growth temperature of 490 °C and exposed to Sb flux for 5 min. Then, a 17 monolayer (ML) AlSb nucleation layer was deposited using a growth rate of 0.36 ML/s. This forms a network of interfacial misfit arrays^14^ at the III-V/Si interface. Next, a 2 μm thick two-step GaSb buffer layer sample was developed using a two-temperature step procedure at 490/515°C without a growth interrupt, leading to a TD density of 2 × 10^8^ cm^− 2^ with no APDs. The specific growth procedure has been described previously^23^. This sample was used as a reference and is depicted in Fig. 1.a. Two DFSL samples were also grown on Si using the two-step GaSb buffer: Superlattice (SL) sample A and B (Figs. 1.b and 1.c respectively). These structures consist of several iterations of AlSb/GaSb SLs grown at 515 °C. The growth rates used for the GaSb and AlSb layers were 0.66 and 0.31 ML/s respectively. SL sample A consists of five superlattices where each one comprises five repeats of the AlSb (10 nm)/ GaSb (10 nm) pair, leading to a structure with a total thickness of 4.2 μm including the underlying GaSb buffer. This standard filter structure was previously used to grow infrared photodetectors on Si^25^. SL sample B is a new design which consists of four SLs each characterized by a different number of iterations of the AlSb/GaSb pair and different layer thicknesses, as described below. The total thickness of SL sample B is 3.7 μm, which is lower compared to that of sample A, and comparable to the thickness of a typical GaAs filter structure^26–28^. Binary AlSb and GaSb semiconductors were chosen for use in the DFSL layer as they share a common group V element leading to reduced atomic segregation and making them less sensitive to growth temperature variations. This simplifies the growth of abrupt interfaces, which is necessary to engineer the strain to effectively block dislocation threading along the growth direction.

**Fig. 2:**
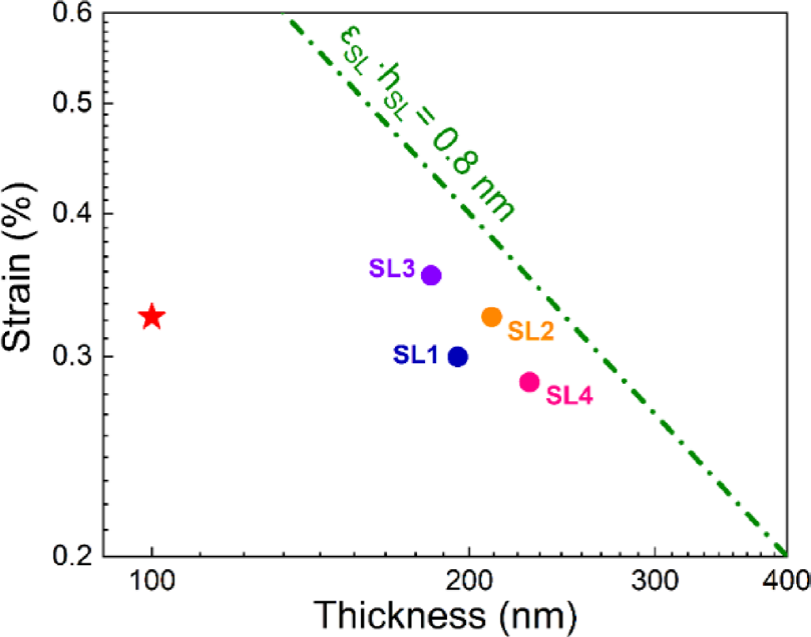
Strain-thickness characteristics of the DFSL structures used in SL sample A (star point) and sample B (dot points). The strain-thickness product limit of 0.8 nm is represented by the green dash-dotted line.

The blocking function of DFSL structures is based on the generation of internal strain fields at the lattice mismatched heterointerfaces which apply a glide force and drive the threading dislocation lines to bow and move parallel to the interface, towards the edge of the sample. The misfit strain is calculated by:

1 $$\:\epsilon\:=\frac{{\alpha\:}_{i}-{\alpha\:}_{s}}{{\alpha\:}_{s}}$$

where α_i_ is the lattice constant of the AlSb layer, and α_s_ is the lattice constant of the underlying GaSb layer. The misfit strain for a single AlSb/GaSb layer is ε = 0.65%. However, as the AlSb layer thickness and/or the number of repeats increases there is a risk of generating new dislocations if the net strain exceeds a critical limit. Therefore, efficient DFSL structures must be carefully designed in order to limit the propagation of preexisting TDs^28^, whilst avoiding the generation of new ones. This has previously been highlighted through the importance of the strain ($\:\epsilon\:$) - thickness (* h*) product^29,30^, which, to prevent the multiplication of defects, must never exceed the limit of

2 $$\:{{\upepsilon\:}}_{\mathrm{S}\mathrm{L}}\cdot\:{\mathrm{h}}_{\mathrm{S}\mathrm{L}}\le\:\:0.8\:\mathrm{n}\mathrm{m}$$

where $\:{{\upepsilon\:}}_{\mathrm{S}\mathrm{L}}$ and $\:{\mathrm{h}}_{\mathrm{S}\mathrm{L}}$ are the total strain and thickness of a DFSL, respectively (see supporting information). As the TD density decreases, the spatial separation between them increases which reduces the probability of a TD interacting with each other. This reduces the efficiency of subsequent DFSL structures. As a result, DFSLs with higher strain fields and thus higher strain-thickness product values need to be used, which have the potential to exceed the 0.8 nm limit and create new defects. To further increase dislocation sweeping by the DFSLs and enhance the motion of dislocations to promote their intersection, and thus reduce their number, higher strain-thickness products are used when proceeding towards the top of the structure. We suggest that by varying the strain-thickness product from a low value close to the III-V/Si interface, to a higher value further away from the interface, an efficient TD filtering structure is created that minimizes the accumulated strain that could otherwise lead to plastic deformation. This hybrid structure was used in SL sample B. Here, the first DFSL ($\:{{\upepsilon\:}}_{\mathrm{S}\mathrm{L}}{\mathrm{h}}_{\mathrm{S}\mathrm{L}}=\:0.584\:\mathrm{n}\mathrm{m})$ will block a vast number of TDs. The second DFSL ($\:{{\upepsilon\:}}_{\mathrm{S}\mathrm{L}}{\mathrm{h}}_{\mathrm{S}\mathrm{L}}=\:0.682\:\mathrm{n}\mathrm{m})$ uses a higher strain-thickness product to further reduce the number of TDs. The third ($\:{{\upepsilon\:}}_{\mathrm{S}\mathrm{L}}{\mathrm{h}}_{\mathrm{S}\mathrm{L}}=\:0.649\:\mathrm{n}\mathrm{m})$ and fourth ($\:{{\upepsilon\:}}_{\mathrm{S}\mathrm{L}}{\mathrm{h}}_{\mathrm{S}\mathrm{L}}=\:0.649\:\mathrm{n}\mathrm{m})$ SLs are also designed to provide higher strain-thickness product values as compared to the first one. These parameters are summarized in Table 1. If all the SLs are placed very close to the 0.8 nm limit, then it is possible that total net strain in the structure might be too high leading to generation of defects. For comparison, a detailed table with the characteristics of the SL structures used for both SL sample A and B is also presented in the Supporting Information (Table S1). Furthermore, as shown in Fig. 2, these values are all close to but below the multiplication limit of 0.8 nm. The strain-thickness product of each of the five identical SLs of sample A is 0.325 nm, which is significantly lower than those used in sample B. Further details of the strain and thickness characteristics of the DFLSs are presented in the Supporting Information.

**Table 1:** Layer thicknesses and strain-thickness products of the four superlattices in SL sample B. The number of AlSb/GaSb pair repeats in each SL, t_i_, are also given.

Characteristics	SL sample B			
	SL_1_	SL_2_	SL_3_	SL_4_
**h**_**GaSb**_ **(nm)**	10.5	10.5	10.5	16
**h**_**AlSb**_ **(nm)**	9	10.5	12.5	12.5
**t**_**i**_	10	10	8	8
**ε**_**SL**_**∙h**_**SL**_ **(nm)**	0.584	0.682	0.649	0.649

## Results and discussion

**Fig. 3:**
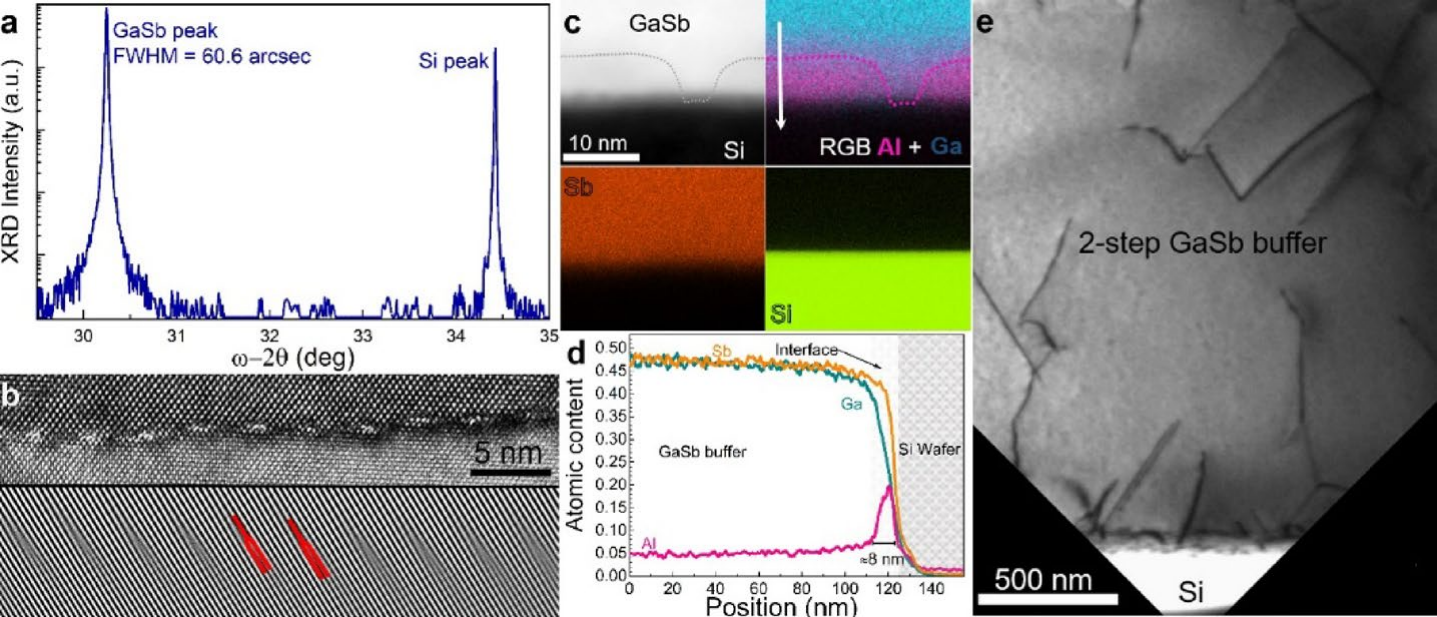
Structural Characterization of the two-step GaSb. (**a**) High resolution X-ray diffraction of the two-step GaSb buffer. (**b**) High resolution [110] TEM image of the III-V/Si interface and Fourier filtered image of the same area obtained using the (1–11) Bragg reflection showing the formation of IMF array with spacing of 3.37 nm. The red lines highlight the atomic layers. (**c**) HAADF-STEM image of the AlSb islands formed at the III-V/Si interface. The magenta, blue, red and light green colors highlight the position of Al, Ga, Sb and Si respectively. (**d**) Elemental variation along the area (white arrow) shown in the RGB image of Fig. 3.c. Further information about the interface elemental map can be found in the Supporting Information. (**e**) Dark field TEM image of the two-step GaSb buffer layer and the TDs originating at the GaSb/Si interface.

Evaluation of the structural characteristics of the two-step GaSb buffer layer was performed using XRD, PL and atomic resolution TEM. As shown in Fig. 3.a, the ω-2θ high resolution XRD pattern of the 004 diffraction peak revealed a narrow full width at half maximum (FWHM) of 60.6 arcsec for the GaSb layer, approximately only two times broader than the Si peak, indicating the excellent crystalline quality of the buffer. Analysis of the diffraction pattern also revealed a residual tensile strain of 0.19% which has been ascribed to thermally induced strain during the cooling process of the sample resulting from the differences in the thermal expansion coefficients^31^ between the GaSb epilayer and Si wafer. The differences between the thermal characteristics of the materials cause different contracting rates during cooling, following the completion of the epilayer growth, and the build-up of thermal shear stress between the two layers resulting in a residual tensile strain in the GaSb epilayer given by^32^:

3 $$\:\varepsilon {_{\:\left\| \right.}} = \int \: _{{T_f}}^{{T_g}}\left[ {\kappa {\:_{GaSb}} - \kappa {\:_{Si}}} \right]dT$$

where κ_GaSb_ and κ_Si_ are the linear thermal expansion coefficients of GaSb and Si respectively. T_g_ is the growth temperature and T_f_ = 25 °C is the final temperature after cooling. The value of 515 °C was used for T_g_, which is the highest temperature used during the growth of the two-step GaSb buffer. While the thermal expansion coefficient of GaSb (κ_GaSb_ = 7.75 × 10^− 6^ °C^-1^^33^ can be considered stable within the temperature range of interest^34^, the corresponding value for Si varies^35^ from approximately 2.5 × 10^− 6^ °C ^-1^ to 4 × 10^− 6^ °C ^-1^ leading to a predicted strain of 0.175% in the GaSb, which is very close to the experimental value (0.19%) obtained from the XRD analysis.

The high-resolution TEM image of Fig. 3.b shows the formation of a regular interfacial misfit (IMF) array at the III-V/Si interface. A (1–11) Fourier filter was applied to highlight the arrangement of the atomic planes around the core of the misfit dislocations. The average IMF dislocation spacing was calculated as 3.37 nm, which is close to the theoretical spacing value of 3.34 nm required for effective release of the misfit strain^36^. Figure 3.c shows the high-angle annular dark-field (HAADF) elemental analysis of an area around the III-V/Si interface where AlSb islands were formed, confirming the excess of Al as well as the absence of Ga within the islands.

The elemental profile shown in Fig. 3.d indicates a high Al concentration in an area with a width of approximately 8 nm. Figure 3.e is a cross-sectional TEM image of the GaSb buffer taken under 002 dark-field conditions, showing the generation of a high density of TDs at the III-V/Si interface. Many of these dislocations are annihilated within the first 700 nm of the GaSb buffer, but a significant number continues to propagate, leading to a surface defect density of 2 × 10^8^ cm^-2^, as measured by ECCI.

**Fig. 4:**
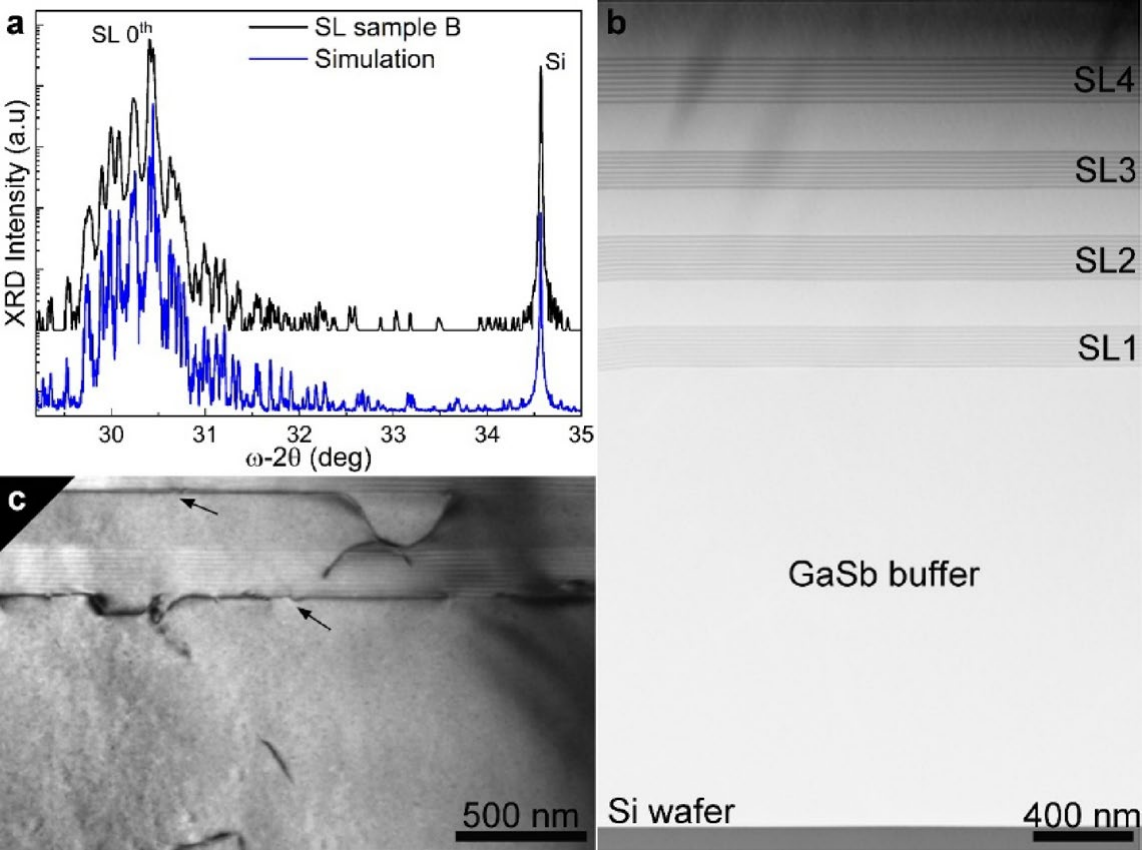
Structural characterization of SL sample B. (**a**) High resolution XRD scan and simulation. (**b**) HAADF STEM image showing the complete structure of the sample. c) High resolution diffraction contrast image of SL1 and SL2, i.e. the two superlattices closest to the Si wafer. The arrows denote the presence of dislocations moving parallel to the image plane.

Examination of the structural quality of the DFSL samples was also carried out using high resolution XRD and TEM. Figure 4 shows the XRD and TEM structural characterization results obtained for SL sample B (XRD experimental data and modelling for SL sample A can be found in Figure S1 of the Supporting Information). Both structures demonstrated well-defined diffraction satellite peaks, which are typical of high-quality superlattices. An increased number of satellite peaks can be observed in the diffraction pattern of SL sample B (Fig. 4.a.) due to the elaborate.

arrangement of the filter layers. Modelling of the experimentally obtained diffraction patterns indicated excellent agreement with the target layer thicknesses. Figure 4.b presents dark field TEM images of sample B showing the individual superlattices of the structure, all demonstrating abrupt interfaces (TEM images of SL sample A are also shown in Figure S2 of the Supporting Information). The HAADF image scales with Z, thus, as expected, threading dislocations climbing from the III-V interface to the DFSLs cannot be identified. A high density of dislocations was.

**Fig. 5:**
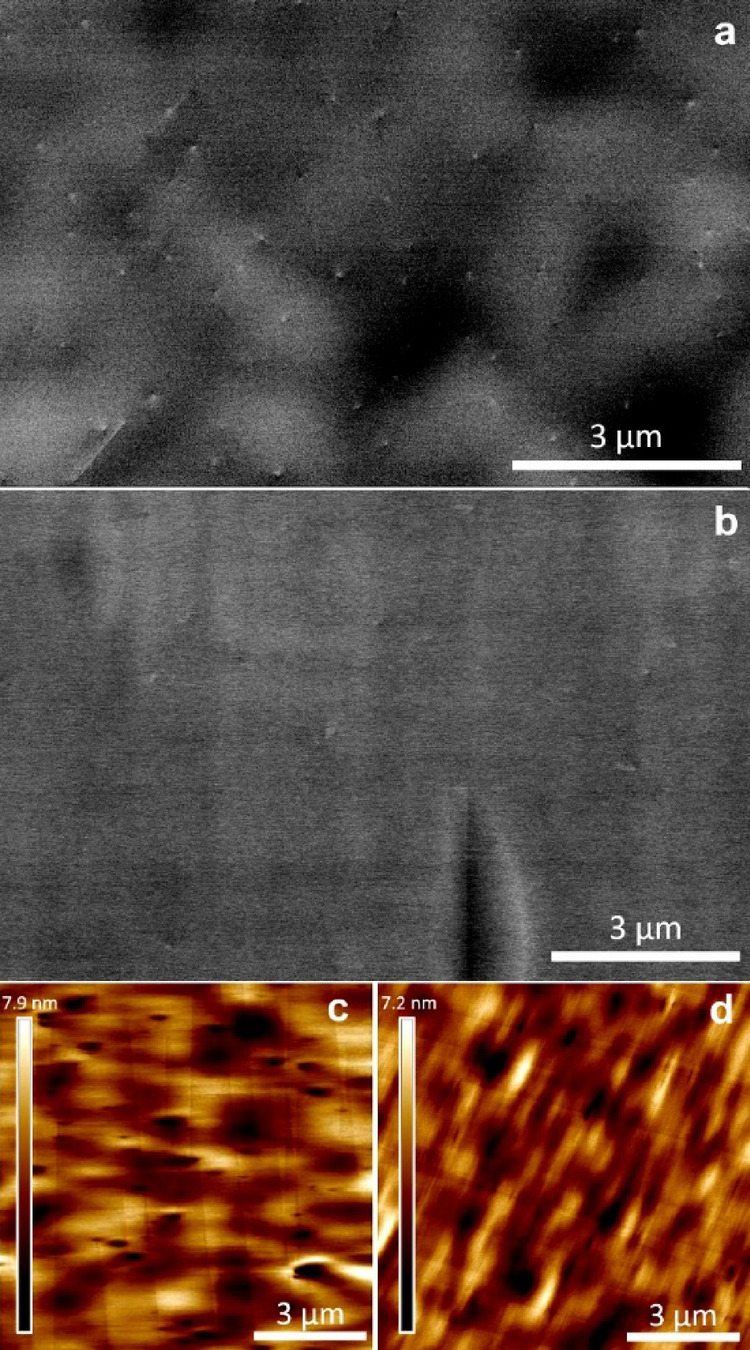
Surface characterization of the two GaSb/AlSb DFSL samples. ECCI surface imaging of SL sample (**a**) A and (**b**) B. AFM surface images of SL sample (c) A and (d) B.

effectively stopped by the first DFSL (Fig. 4.c) as many are encouraged to propagate laterally, along the strained SL interfaces of the filter layers, rather than vertically to overlying layers. However, a significant number of TDs pits were still identified at the top interface of the final superlattice of the structure. Figures 5.a and b are ECCI surface images of the top surfaces of SL sample A and SL sample B, respectively. Threading dislocations as well as planar defects can be identified on the surface of both samples. SL sample A’s relatively simple repeating superlattice structure of 10 nm GaSb/ 10 nm AlSb resulted in a surface defect density of 3 × 10^7^ cm^− 2^, which is approximately an order of magnitude lower than that obtained for the two-step buffer. AFM surface imaging of the SL sample A revealed a root mean square (RMS) roughness of 1.2 nm. For SL sample B the surface defect density was significantly lower at 6 × 10^6^ cm^− 2^, indicating that despite its lower overall thickness, a filter structure with increasing SL strain-thickness product values, following the strain-thickness limitation rule, leads to a substantially lower surface defect. To the authors’ best knowledge this value is the lowest reported for III-Sb semiconductors heteroepitaxially grown on Si to date. Comparable defect densities have been reported previously only for GaAs on Si samples^26,28^. Furthermore, the RMS surface roughness of SL sample B was measured to be 1.15 nm, which is similar to that obtained for SL sample A. This suggests that the planar defects are the main cause of the surface roughness, in agreement with previous publications^34^. The formation of crosshatched patterns was observed in both the ECCI and AFM images shown in Fig. 5. This is a well-known phenomenon in semiconductors. This strain originates from plastic relaxation and the formation of misfit dislocations. Local strain concentrations surrounding the network of IMF dislocations lead to enhanced growth rates around the dislocation areas, all resulting in the formation of well-oriented crosshatch patterns^37^.

**Fig. 6:**
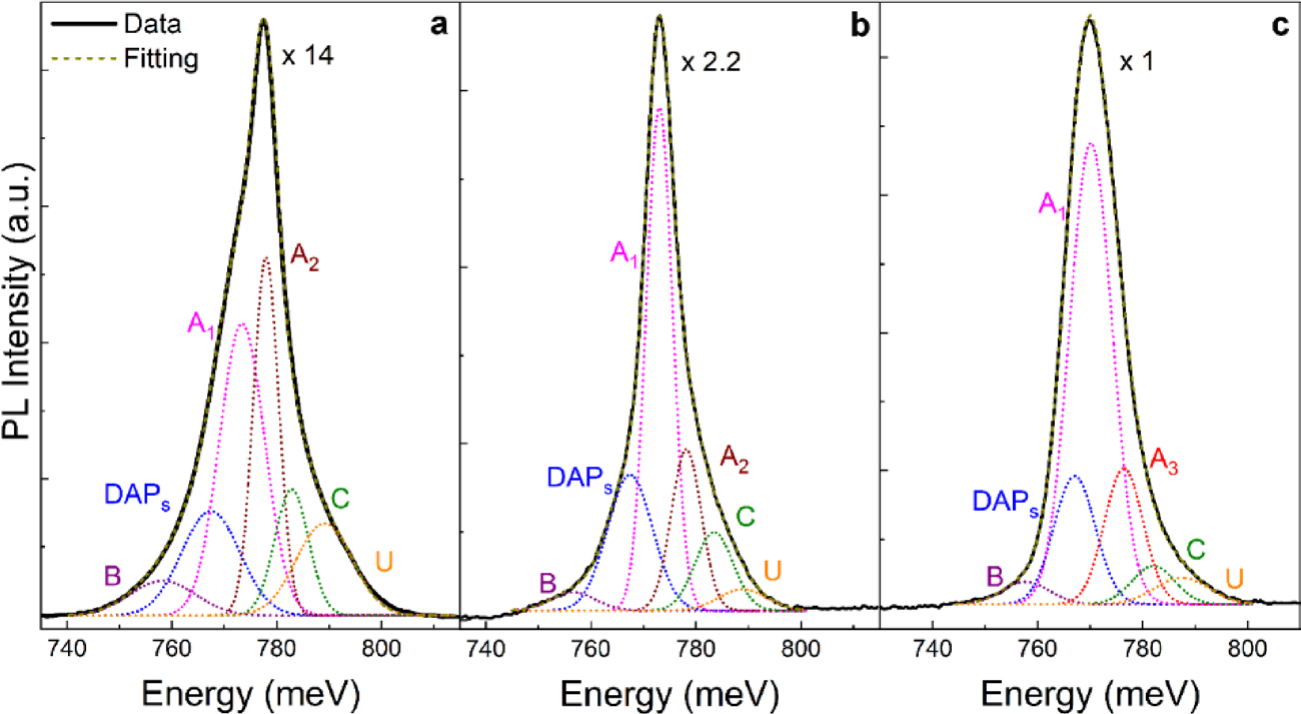
Low temperature PL (4 K) characterization. Results obtained for (**a**) the two-step GaSb buffer, (**b**) SL sample A and (**c**) sample B. The PL intensity of SL sample B is roughly 2.2 and 14 times more intense than SL sample A and the two-step buffer sample respectively. The PL peaks were deconvoluted and fitted using a Gaussian distribution.

The samples were investigated further using 4.2 K photoluminescence measurements. This temperature was chosen to reduce non-radiative Auger recombination and to allow detection of defect-related trap states to assess the material quality. Emission related to free or bound excitons in GaSb was not observed, however all three samples exhibited a similar set of PL emission peaks, as shown in Fig. 6. Two peaks at 773 and 778 meV, denoted A_1_ and A_2_ respectively, are commonly attributed to native acceptor levels in GaSb^38,39^. These peaks exhibited only a very small blueshift (≤ 0.5 meV) with increasing laser excitation power (see Figure S3 in Supporting Information), which is characteristic of transitions from the conduction band to acceptor levels^38^.

A third peak due to donor-acceptor pair transitions, denoted as DAP_S_, was identified at around 767 meV, which is due to recombination between shallow and deep defects^40^. Transition C, which is present at higher energies of 780 to 783 meV is related to a donor-to-carbon pair recombination arising from residual carbon impurities in the MBE system following the Si wafer outgassing procedure^41^. Transition B (756 to 758 meV) is related to recombination from the conduction band to a deep native acceptor state^42^. A high energy peak U is observed around 789 meV; however, as stated in previous report^39^, it could not be ascribed to a specific transition. The PL emission from the two-step GaSb buffer sample, shown in Fig. 6.a, is dominated by the two native acceptor recombination’s with transition A_2_ having the main contribution. For both SL sample A and B, peak A_1_ is the dominant transition. It is interesting to note that the same two native acceptor-related peaks could be observed for the two-step GaSb buffer and SL sample A, whereas for SL sample B a third native acceptor-related peak^38^, A_3_, was observed demonstrating a blue shift of 2 meV with laser power (see Figure S4 in Supporting Information) and a red shift of 1meV with temperature, (see Figure S6 in Supporting Info) behaviour characteristic to donor-acceptor recombination^43^. These results suggest a partial shift of the native acceptor level possibly by the strain potential of the threading dislocations^44^. Reducing the threading dislocation density below 10^7^ cm^− 2^ could eliminate the deformation potential leading to a single band-to-native acceptor related transition peak for sample B. Furthermore, a reduction in the strain associated with the TDs may allow transitions between the native donor and acceptor levels formed by gallium vacancy-gallium antisite complexes, resulting in the A_3_ peak. Small variations of the peak energies were observed for each sample which are probably caused by differences in the residual strain. The PL intensity of SL sample B is about 2.2 and 14 times stronger compared to that of SL sample A and the buffer sample respectively. Furthermore, the maximum PL intensity is only 1.4 times less intense than that obtained for the 2 μm thick homoepitaxial GaSb control sample (see Supporting Information and Figure S5). The lower PL intensity of the two-step buffer and the SL sample A can be related to their higher surface defect density and thus an increased number of non-radiative recombination centers. Furthermore, a significant decrease in the carbon related peak was observed for the superlattice samples. This suggests carbon contamination decreases in the growth chamber for thicker structures that have longer growth periods.

**Fig. 7:**
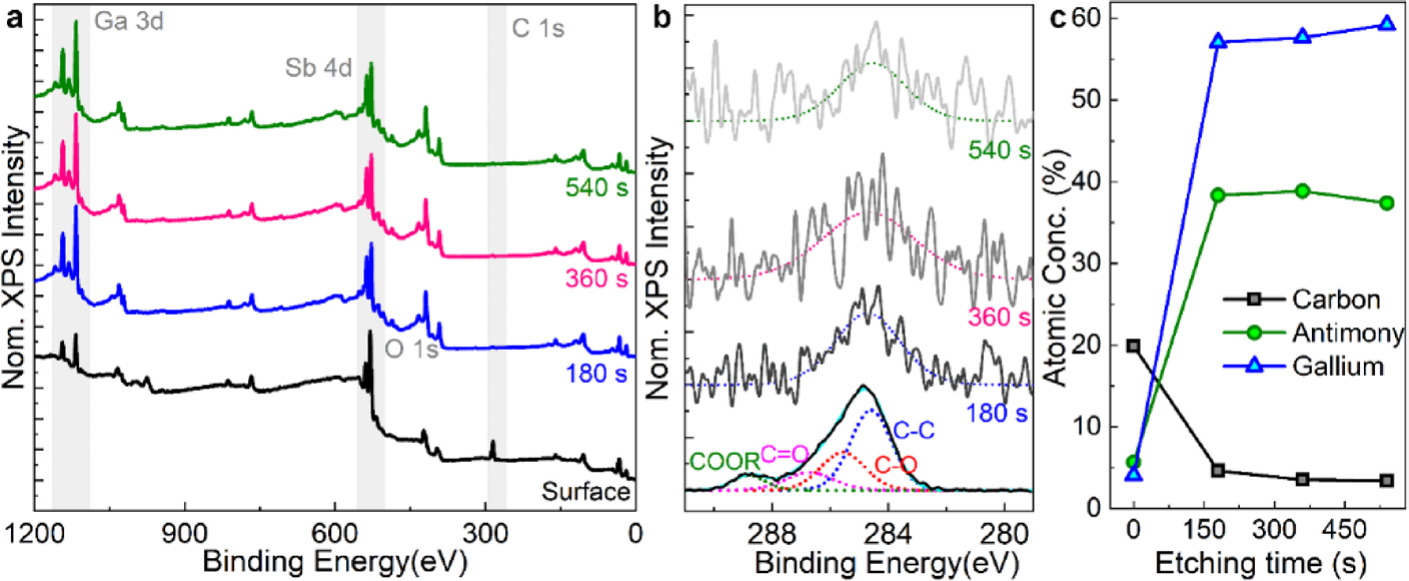
Ion sputtering and XPS analysis of the two-step GaSb buffer. (**a**) A series of wide binding energy XPS spectra and (**b**) C 1s core level spectra obtained at the surface and following each Ar ion sputtering step. It is estimated that after 540 s the etching depth is approximately 90 nm. (**c**) XPS depth profile obtained using a 4 keV ion incident beam. It should be noted here that the carbon obtained following the first etching step was higher than that obtained after 360 and 540 s due to potential carbon transfer during the Ar^+^ etching.

Finally, XPS surface characterization alongside Ar^+^ ion sputtering etching was used to evaluate the chemical composition of the surface and confirm the presence of residual carbon atoms in the samples. Figure 7 shows the XPS results obtained for the two-step buffer sample etched with Ar^+^ ions for different durations. The wide binding energy (BE) spectrum obtained from the surface of the as-grown sample was characterized by strong carbon, Ga, Sb and oxygen related peaks as shown in Fig. 7.a. High resolution analysis indicated that the carbon peak is attributed to adventitious carbon (Fig. 7.b) formed on the surface of the sample due to atmospheric exposure and storage, while the Ga 2p and Sb 3p peaks were dominated by the presence of native oxides^45,46^(Figure S7). Following the Ar^+^ ion bombardment, mostly Ga and Sb related peaks were identified in the wide BE spectra (Fig. 7.a), while the presence of the GaSb compound was confirmed by the binding energy of the Ga 2p and Sb 3p peaks (Figure. S7), which were in excellent agreement to the literature^47^. High resolution spectra of the C 1s peak was also recorded following each ion etching step and carbon traces were identified even after 540 s of etching. For an etching time longer than approximately 200 s it is expected that carbon related to surface oxides will be completely removed^48^. Thus, the identification of carbon traces confirms the presence of carbon residuals in the sample and supports the earlier assignment of the C transition in the PL spectrum as being carbon related. Carbon in GaSb acts as an acceptor^49^ when substituting Sb. The local increase of electron density is expected to affect the Coulomb interactions and shift the binding energy towards lower values, resulting in a shift of the 1s carbon peak towards lower energies^50^ as compared to the main carbon peak (284.6 eV). The C 1s peak of the surface following the ion bombarding for 540 s was located at 284.5 eV, which is 0.1 eV lower than peak energy of the main carbon peak. However, it should be noted that identifying the peak position is difficult due to the small signal intensity and the high background noise of the peak, as shown in Fig. 7.b. Furthermore, the Ga and Sb binding energies are only expected to be affected by large amounts of carbon, such as in cases of intentional doping^51^. Surface stoichiometric analysis was obtained for each ion bombardment step, and the results are shown in Fig. 7.c. The surface of the as-grown GaSb could be considered stoichiometric. However, the carbon (~ 20%) and oxygen (~ 70% not shown) contaminant atoms dominate the surface of the sample. When ion bombardment was used, the treated surface was found to be Ga-enriched due to the preferential.

loss of Sb atoms during sputtering^48^, while the Ga 2p3/2 and Sb 3d5/2 core-shell peaks (Figure S7) related to the bonds formed between the Ga and Sb atoms of GaSb were centered at 1116.8 eV and 528 eV, respectively. Similar results were also obtained for SL sample B (Figure S8). Furthermore, it should be noted that the identified amount of residual carbon within the structure following 360 s of etching was approximately 3 times lower compared to that obtained for the two-step GaSb buffer, confirming the reduction of carbon contaminants when prolonged growth procedures are used.

## Conclusion

In conclusion, AlSb/GaSb superlattice structures were grown on silicon substrates using a two-temperature-step GaSb buffer and interfacial AlSb islands. The large lattice constant mismatch at the Si/III-V semiconductor interface was successfully relieved via the formation of a network of interfacial misfit arrays. However, a significant number of threading dislocations remained and reached the surface of the GaSb buffer. The use of five identical AlSb/GaSb dislocation filters reduced the surface dislocation only by an order of magnitude to ~ 10^7^ cm^− 2^. To further promote dislocation annihilation and decrease their density, a novel AlSb/GaSb filter structure consisting of four unique superlattices was designed. In this sample, the thickness of the AlSb/GaSb pairs in each subsequent superlattice is greater than the last, whilst the strain-thickness product is always lower than the multiplication limit of 0.8 nm. This design successfully reduced the surface defect density down to 6 × 10^6^ cm^− 2^, which, to the best of the authors knowledge, is the lowest value ever reported for III-Sb deposited on Si. The reduction of threading dislocations within the filter structures was also confirmed by low temperature PL analysis. The maximum PL intensity obtained from the novel SL sample was approximately 14 times stronger than that of the GaSb/Si sample with no SL. Furthermore, analysis of PL results indicated the presence of carbon within the samples incorporated into the structure was a result of the in situ wafer cleaning. The presence of carbon was also confirmed using surface ion sputtering and XPS compositional analysis. However, the DFSL structures were able to reduce the carbon residuals. Reducing the dislocation density to even lower levels (towards 1 × 10^6^ cm^− 2^) may be possible by further optimizing the design of the DFSL structures which will be the subject of our future work. Our results open the pathway to high quality GaSb epilayers on silicon to enable the integration of room efficient photonic and electronic devices on Si.

## Methods

### Growth technique

All samples were grown using a solid source Veeco GENxplor Molecular Beam Epitaxy (MBE) system operated at cryogenic temperature and ultra-high vacuum conditions. The system is equipped with a dual filament gallium source, a valved cracker Mark V 200 cc antimony cell and a SUMO dual filament aluminum cell.

### Si wafer surface oxide desorption

The thermal cleaning technique of the Si wafers’ surface consists of two annealing steps without the use of any reducing gas. During the first step the wafers were heated up at 600 °C within the pre-growth chamber. Then they were cooled down to 200 °C and transferred to the growth chamber. The second thermal cleaning occurred within the growth chamber where it underwent several annealing temperature cycles with the actual wafer temperature progressively increasing up to approximately 1000 °C, followed by cooling down to the growth temperature.

### Structural, optical and surface characterization

Transmission electron microscopy (TEM) images were collected using a Philips CM 200 and a JEOL 2100 EX TEM system operating at 200 keV. A Zeiss Gemini scanning electron microscope (SEM) equipped with a solid-state backscatter detector operating at 20 kV was used to collect the electron channeling contrast images (ECCI). A Multimode Atomic Force Microscope (AFM) in tapping mode connected to a Nanoscope 8 controller was also used to perform AFM surface characterization over a scanning area of 10 μm × 10 μm. High resolution X-ray diffraction (XRD) characterization was carried out using a Bruker AXS D8 Discover X-ray diffractometer. XRD simulations were generated using RADS Mercury software. Low temperature photoluminescence (PL) measurements were performed using a 532 nm laser and an excitation power of 210 mW. Optical fibers were used for laser light delivery and collection of sample emissions which were detected using a spectrometer and Peltier-cooled InGaAs array detector. X-ray photoelectron spectroscopy (XPS) spectra were recorded using a Kratos AXIS Ultra^DLD^ XPS system equipped with a hemispherical analyser and an aluminium X-ray source. Wide binding energy (BE) XPS spectra were obtained using a pass energy of 160 eV and high-resolution spectra were acquired using 20 eV. The pressure of the testing chamber was of the order of 10^− 9^ Torr during the measurements. An Ar^+^ ion gun with a beam energy of 4 keV was used for the ion sputtering. The spectra obtained for the as-grown and Ar^+^ sputter surface were corrected to the C 1s (284.6 eV) and Sb 3d (528.2 eV) peak for GaSb respectively. The Shirley background correction was employed while peak fitting was performed assuming a Lorentzian-Gaussian (30:60) distribution.

## Supplementary Information

Below is the link to the electronic supplementary material.

Supplementary Material 1

## Data Availability

The datasets generated and analysed during the current study are available in the [Pure] repository, [https://dx.doi.org/10.17635/lancaster/researchdata/722]
