# Supplementary material for: Threading dislocation reduction in heteroepitaxial GaSb based superlattices grown on silicon
Source: Sci Rep. 2026 Jun 3;16:25327. doi: 10.1038/s41598-026-55373-4 (PMC13473081; doi:10.1038/s41598-026-55373-4)
Supplement: Supplementary file 1 [file 41598_2026_55373_MOESM1_ESM.docx]

Supporting Information

**TITLE. Threading Dislocation Reduction in GaSb based Superlattices Heteroepitaxially Integrated on Silicon**

**AUTHORS. Evangelia Delli^1^*, Mathew Bentley^1,2^, Niall Mulholland^1^, Peter D. Hodgson^2^, Maria de la Mata^3^, Sergio I. Molina^3^, Richard Beanland^4^, Magnus C. Wagener^5^, Johannes R. Botha^5^, Andrew R. J. Marshall^2^, and Peter J. Carrington^1^***

^1^School of Engineering, Lancaster University, Bailrigg, Lancaster, LA1 4YW, UK

^2^Department of Physics, Lancaster University, Bailrigg, Lancaster, LA1 4YB, UK

^3^Department of Material Science and Metallurgic Engineering, University of Cadiz, Puerto Real, Cadiz, 11510, Spain

^4^Physics Department, University of Warwick, Coventry, CA4 tAL, United Kingdom

^5^Department of Physics, Nelson Mandela University, Gqeberha, 6031, South Africa

NUMBER OF PAGES. 15

NUMBER OF FIGURES. 8

NUMBER OF TABLES. 2

**Design of GaSb/AlSb dislocation filters**

The strained interfaces of DFSL structures can significantly enhance the motion and glide of dislocations, thus promoting the reaction and bowing of the dislocation line^1^. However, the reduction or multiplication of TDs depends upon the misfit strain of the layers. For alternative layers with an interfacial mismatch up to ~9% at the interface, the epilayers are expected to be elastically and uniformly strained to accommodate the strain and are free of any newly generated dislocations. For higher misfit values or very thick layers, the strain is elastically accommodated until the epilayer reaches the Matthews critical thickness, beyond which generation of dislocations is energetically favorable to reduce the strain^2^. In 1997 Dunstan^3^ suggested that heteroepitaxial layers with a specific strain ($\varepsilon$) – thickness ($h$) product value demonstrated excellent crystalline structure and limited defect density. Based on experimental data the strain-thickness product limit of

$$\varepsilon\cdot h\leq0.8 nm (1)$$

was considered the optimum to obtain the so-called “perfect” samples. The misfit strain is calculated as

$$\varepsilon=\frac{\alpha_{i}-\alpha_{s}}{\alpha_{s}}100\% (2)$$

where $\alpha_{i}$ is the lattice constant of the layer and $\alpha_{s}$ the lattice constant of the underlying substrate or epitaxial layer.

For the AlSb/GaSb system, the strain of the layers is approximately |0.65| % which is well below the limit of 2% for elastic growth and limited formation of new defects. SL sample B was grown so that the thickness of the layers, h_j,i_, where i=1,2,3,4 the number of the corresponding SL and j=AlSb or GaSb, was kept well below the Matthews critical thickness which is approximately 20 nm to avoid the generation of new defects. Also, aiming to enhance the defect motion at each following SL step, the four DFSLs were configured in a way that starting from the SL placed right above the thick GaSb buffer and going towards the top of the structure the thickness of the layers and/or the thickness of the GaSb/AlSb pair of layers, h_pl,i_, increases for each subsequent SL. Increasing the film thickness is expected to enhance the interaction strength between threading dislocations while reducing the strain hardening^4^.

The thickness of the GaSb/AlSb pair of layers is given by

$$h_{pl,i}=h_{GaSb,i}+h_{AlSb,i} (3)$$

where h_GaSb,i_ and h_Alsb,i_ are the thicknesses of the GaSb and AlSb layers. Furthermore, each superlattice was considered to act as a single layer of thickness

$$h_{SL,i}=h_{pl,i}\cdot t_{i} (5)$$

with strain

$$\varepsilon_{SL,i}=\frac{\left( \frac{\alpha_{\mathrm{GaSb}}\cdot h_{GaSb,i}+\alpha_{\mathrm{AlSb}}\cdot h_{AlSb,i}}{h_{pl,i}} \right)-a_{s}}{a_{s}} (6)$$

where $t_{i}$ is the number of iterations and $\alpha_{\mathrm{GaSb}}$ and $\alpha_{\mathrm{AlSb}}$ are the lattice constants of GaSb and AlSb respectively. The thickness of the layers and pair of layers as well as the number of iterations of the pair of layers was chosen so that the total superlattices thickness and strain of each SL does not exceed the multiplication limit of $\varepsilon_{SL,i}\cdot h_{SL,i}\leq0.8 nm$ to avoid the formation of additional dislocations. Thus, the number of periods was reduced from 10 for the first two SLs down to 8 for the third and fourth SL as these have thicker superlattice layers. Furthermore, this limitation alongside the need for increasing the thickness of the layers or/and the GaSb/AlSb pair of layers for each subsequent SL resulted in SL2 having the higher strain-thickness product. SL 3 and SL 4 have a strain thickness product higher than that of SL1 but approximately 1.05 times lower compared to that of SL2. Table S1 summarizes the strain and thickness characteristics of the GaSb/AlSb SLs used in SL sample B as compared to SL sample A.

**Table S1.** Comparison of the strain-thickness characteristics of the four individual superlattices of the two superlattice structures. The same sample naming is used as in the main text.

|  | **SL sample B** | | | | **SL sample A** |
| --- | --- | --- | --- | --- | --- |
| **Characteristics** | **SL_1_** | **SL_2_** | **SL_3_** | **SL_4_** | **SL_1-5_** |
| **h_GaSb_ (nm)** | 10.5 | 10.5 | 10.5 | 16 | 10 |
| **h_AlSb_ (nm)** | 9 | 10.5 | 12.5 | 12.5 | 10 |
| **ε_AlSb_ (%)** | 0.649 | 0.649 | 0.649 | 0.649 | 0.649 |
| **h_pl_ (nm)** | 19.5 | 21 | 23 | 28.5 | 20 |
| **t_i_** | 10 | 10 | 8 | 8 | 5 |
| **h_SL_ (nm)** | 195 | 210 | 184 | 228 | 100 |
| **ε_SL_ (%)** | 0.3 | 0.325 | 0.353 | 0.285 | 0.325 |
| **ε_SL_∙h_SL_ (nm)** | 0.584 | 0.682 | 0.649 | 0.649 | 0.325 |

**Structural characterization of GaSb/AlSb dislocation filter structures**

**High resolution X-ray diffraction.** Both AlSb/GaSb SL sample A and B demonstrated narrow and well-formed satellite peaks indicating the excellent structural quality of the superlattices (Fig. S1). Modelling of the XRD data of both structures using RADS Mercury software revealed excellent agreement to the target layer thickness.

**Figure S1.** High resolution XRD pattern and simulation of both Sb-based filter structures.

**Transmission electron microscopy images.** TEM images from SL sample A are shown in Fig. S2.a. The structure demonstrated well-defined superlattice layers in agreement with the XRD pattern. Most of the threading dislocations are stopped by the first dislocation filter. The remaining TDs are occasionally forced to propagate perpendicular to the growth direction as shown in Fig. S2.b.

**Figure S2**. (**a**) Dark field TEM image showing the five identical superlattices of SL sample A grown on Si. (**b**) Bright field image of the top two superlattices. Tips of the threading dislocations moving perpendicularly to the image plane can be clearly seen.

**Elemental map around the III-V/Si interface.** The chemical composition of the two-step GaSb buffer sample at the interface was investigated to confirm the nature of the observed islands shown in Figure S3.a. 2D maps of the elemental constituents by means of EDX were obtained. An RGB composition image of Al

**Figure S3.** (**a**) HAADF image of the GaSb/Si interface confirming the formation of the AlSb islands. (**b**) RGB composition image of Al and Ga. (**c**) EDX spectra obtained from the Si wafer and an area around the GaSb/AlSb islands interface as indicted by the arrow in Figure S3.b. Atomic content map of the III-V/Si interface (**d**) before and (**e**) after the background correction.

and Ga signal is shown in Figure S3.b showing the Al placed solely between the GaSb/Si interface. The white arrow shows the area across which the integrated profile shown in Figure S3.c was taken. The Al-rich region extends about 8 nm axially from the Si substrate, in good agreement with the observed height of the AlSb islands. Due to the nature of the sample and the experimental set-up, a strong silicon background signal detected as indicated from the EDX spectra shown in Figure 3S.c. As a result, the initial quantification underestimates the content of the remaining elements, Al, Ga and Sb, result in lower content percentages compared to the expected values, as shown in Figure S3.d. Following normalization of the spectra to remove the background, the Ga and Sb atomic content is close to 0.5. Regarding the area where the presence of the AlSb islands is expected, it must be noted that, according to the EDX results, an Al-rich AlGaSb area is identified instead of pure AlSb. This might be related to atoms exchanged during the formation of the islands which could potentially resulted in regions with high Al-content, reaching AlSb composition, while other locations would show less Al content.

**Low temperature photoluminescence**

The low temperature, power-depending laser photoluminescence spectra measured from all three heteroepitaxial samples is shown in Fig. S4. It is noted that for the two-step GaSb buffer and SL sample A peaks A_1_ and A_2_ centered at around 773 and 778meV (using 210 mW) demonstrated a negligible shift with increasing laser excitation power. For SL sample B, a third peak, A_3_, was identified related to transition from native GaSb defects. This peak demonstrated a blue shift of around 1.8 meV with increasing the laser power from 30 to 210 meV (x7 increase) and a shift of 2.1 meV when increasing from 3 to 210 meV (x 70 increase).

The low temperature photoluminescence spectra of a homoepitaxially-grown GaSb control sample is shown in Figure S5 (a). In addition to peaks related to native defects, several excitonic emission peaks^5^ (FE, BE1, BE, BE3 and BE4) were identified, significantly changing the shape of the emission of the homoepitaxial

**Figure S4**. Low temperature, power dependent PL (4K) results obtained for (**a**) the two-step GaSb buffer, (**b**) SL sample A and (**c**) sample B. The same color annotation was used for the various transitions peaks as the one presented in Figure 6 in the main text.

**Figure S5**. Low temperature PL (4K) results obtained for the homoepitaxial GaSb layer.

GaSb sample compared to the heteroepitaxial two-step GaSb buffer and the SL samples. It should be noted that a single peak was used to fit the transitions related to the GaSb native defect transitions. It is also important to mention that transition C wasn’t observed for this sample confirming the fact that the origin of the peak is related to the Si wafer outgassing for the samples grown on Si.

**Temperature dependance photoluminescence**

The photoluminescence spectra measured from sample B at 4, 25, 60 and 77 K are shown in Figure S6. Peak A_1_ and A_3_ show a small red shift of approximately 2 and 1 meV with increasing temperature due to the bandgap shrinkage, as expected for native acceptor related peaks^6^. A small shift was also observed for DAP peak ($\sim4.5$ meV) confirming its origin from donor-acceptor pair transitions. The peaks also became less intense due to the thermal ionization of the acceptor stated. The localized carbon acceptor level related peak remained near the same energy and quenched slower than peak A_1_, as the carbon acceptor level is deeper compared to the native acceptor levels. Interestingly, with the increase of temperature and the thermal quenching of the dominant radiative native acceptor levels, a low intensity peak related to excitons bound to neutral acceptor states^7^, BE4, was also observed at 60 and 77 K. As acceptor trapping decreases with temperature and the dominant acceptor related peaks quench the bound excitons peak becomes stronger^8^.

**Figure S6**. PL results obtained for sample B at a. 4K, b. 25K, c. 60K and d. 77K. The gray dashed lines are guides to the eyes showing the small red shifts obtained for the A_1_, A_3_ and DAP peak. All data were using a laser power of 210mW.

**Summary of the surface characterization and PL results**

Table S2 presents an overview of the structural and optical characterization results obtained for three samples grown on silicon, namely the two-step GaSb buffer, SL sample A and B. The SL structure grown using the strain-thickness limitation rule demonstrated the lowest surface defect density and the strongest PL intensity due to the effective threading dislocation blocking at the interfaces of the superlattices.

**Table S2**. Characterization results of the three heteroepitaxial samples.

| Structure | Surface threading dislocation density (cm^-2^) | RMS surface roughness (nm) | PL intensity at 4K (a.u.) | Norm. PL Intensity at 4K |
| --- | --- | --- | --- | --- |
| Two-step GaSb buffer^9^ | 2 x 10^8^ | 2 | 2.0 x 10^4^ | 0.0741 |
| AlSb/GaSb SL sample A | 3 x 10^7^ | 1.2 | 1.2 x 10^5^ | 0.444 |
| AlSb/GaSb SL sample B | 6 x 10^6^ | 1.15 | 2.7 x 10^5^ | 1 |
| Homoepitaxial GaSb buffer | - | - | 3.7 x 10^5^ | 1.4 |
|  |  | |  |  |

**X-rays Photoelectron Spectroscopy**

XPS was employed to investigate the composition of the surface of the two-step GaSb buffer and SL sample B. XPS depth profiling with the help of Ar^+^ ions was also performed to enable examination of the chemical composition of the samples below the first few surface atomic layers which are highly affected by exposure to atmospheric contamination. Figure S7 shows the high resolution XPS Sb 3d and Ga 2p core level spectra of the as-grown and Ar^+^ ion bombarded surface of the two-step GaSb buffer. The fitting of both spectra indicated the presence of native Sb- and Ga-oxides prior to the Ar^+^ ion surface etching. In particular, the O 1s and Sb 3d spectra regions (Fig. S7.a) depicted the presence of Sb_2_O_3_ on the surface of the sample. The corresponding Sb3d_5/2_ and Sb3d_3/2_ peaks are centered at 530 eV and 539.2 eV respectively and are attributed to the bonds formed between oxygen and Sb. Peaks related to the bonds formed between the carbon and oxygen atoms of the surface alongside the Sb3d_5/2_ (527.4 eV) and Sb3d_3/2_ (536.8eV) peaks of GaSb were also present. Following 360 seconds of Ar^+^ etching no signal of oxygen-related peaks was detected. It is expected that after 360 second approximately 60 nm of GaSb has been removed. Figure S7.b also shows

**Figure S7.** High resolution XPS spectra of (a) the Sb 3d and (b) Ga 2p transition peaks of the as-grown and Ar^+^ ion treated GaSb surface (360 seconds sputtering time) of the two-step GaSb buffer sample.

**Figure S8.** High resolution XPS spectra of (a) the C 1s, (b) the Sb 3d and (c) Ga 2p transition peaks of the as-grown and Ar^+^ ion treated surface (360 seconds sputtering time) of SL sample B.

the Ga 2p core level spectra of the surface and ion treated buffer sample. The peaks obtained from the non-treated surface of the sample are shifted by approximately 0.5 eV towards higher binding energies compared to those obtained following the Ar^+^ treatment due to the presence of the Ga oxides. Additionally, the Ga2p_3/2_ and Ga2p_1/2_ peaks obtained following each ion etching step were all centered at 1117.1 eV and 1143.9eV respectively, indicating the pure bonding of the Ga atoms to Sb^10^. These results agree with those reported previously for as received and ion treated GaSb wafers^11^. The GaSb/AlSb SL sample B was also tested using XPS and the results were similar to those obtained for the GaSb buffer as shown in Figure S8. The carbon atomic concentration was reduced from approximately 17% at the surface of the sample down to 1% after 360 seconds of etching, which is approximately 3 times lower compared to that obtained for the GaSb buffer, indicating the decrease of the residual carbon atoms in the growth chamber as the growth proceeds.

AUTHOR INFORMATION

Corresponding Author

*E-mail: evdelli@physics.auth.gr

*E-mail: p.carrington@lancaster.ac.uk.

REFERENCES

(1) Ward, T.; Sánchez, A. M.; Tang, M.; Wu, J.; Liu, H.; Dunstan, D. J.; Beanland, R.; Design rules for dislocation filters, J. Appl. Phys., 116, 063508, 2014.
(2) Romanov, A. E.; Pompe, W.; Mathis, S.; Beltz, G. E.; Speck, J. S.; Threading dislocation reduction in strained layers, J. appl. Phys. 85, 1, 182, 1999.
(3) Dunstan, D. J.; Review: strain and strain relaxation in semiconductors, L. Mater. Sc.: Mat. Electr., 8, 337-375, 1997.
(4) Fertig III, R. S.; Baker S. P.; Dislocation dynamics simulation of dislocation interactions and stresses in thin films, Acta Materialia, 58, 5206-5218, 2010.
(5) Ivanov, S. V.; Altukhov, P. D.; Argunova, T. S.; Bakun, A. A.; Budza, A. A.; Chaldyshev, V. V.; Kovalenko, Y. A.; Kop'ev, P. S.; Kut, R. N., Meltser, B. Y.; Ruminov, S. S.; Shaposhnikov, S. V.; Sorokin, L. M.; Ustinov, V. M.; Molecular beam epitaxy growth and characterization of thin (<2 mu m) GaSb layers on GaAs (100) substrates, Semiconducd. Sci. Technol., 8, 347, 1993.
(6) Wu, M. C.; Chen, C. C.; Photoluminescence of highquality GaSb grown from Ga and Sbrich solutions by liquidphase epitaxy, J. Appl. Phys. 72, 4275, 1992.
(7) Lee, M.; Nicholas, D. J.; Singer, K. E.;, Hamilton, B.; A photoluminescence and Hall-effect study of GaSb grown by molecular beam epitaxy, J. Appl. Phys. 59, 2895–2900, 1986.
(8) Park, J. H.; Lee, T. K.; Noh, Y. K.; Kim, M. D.; Oh, E.; Temperature and excitation power dependence of photoluminescence from high quality GaSb grown on AlSb and GaSb buffer layers, J. APPL. PHYSICS 105, 043516, 2009.
(9) Delli, E.; Letka, V.; Hodgson, P. D.; Repiso, E.; Hayton, J. P.; Craig, A. P.; Lu, Q.; Beanland, R.; Krier, A; Marshall, A. R. J.; Carrington, P. J.; Mid-Infrared InAs/InAsSb Superlattice nBn Photodetector Monolithically Integrated onto Silicon, ACS Photonics, 6, 2, 538–544, 2019.
(10) Gallium Spectra – GaSb – single crystal. The International XPS Database 1. https://xpsdatabase.com/gallium-spectra-gasb/
(11) Vincent Crist, B.; Handbooks of Monochromatic XPS Spectra: Volume 3 – Semiconductors, XPS International LLC, 2019.
